# A Method of Short Text Representation Fusion with Weighted Word Embeddings and Extended Topic Information

**DOI:** 10.3390/s22031066

**Published:** 2022-01-29

**Authors:** Wenfu Liu, Jianmin Pang, Qiming Du, Nan Li, Shudan Yang

**Affiliations:** 1 State Key Laboratory of Mathematical Engineering and Advanced Computing, Zhengzhou 450001, China; wenfugogo123@163.com (W.L.); qimingducst@163.com (Q.D.); linan_happy@126.com (N.L.); yangshudan1111@163.com (S.Y.); 2 State Key Laboratory of Complex Electromagnetic Environment Effects on Electronics and Information System, Luoyang 471003, China

**Keywords:** short text representation, topic information, word embeddings, information fusion

## Abstract

Short text representation is one of the basic and key tasks of NLP. The traditional method is to simply merge the bag-of-words model and the topic model, which may lead to the problem of ambiguity in semantic information, and leave topic information sparse. We propose an unsupervised text representation method that involves fusing word embeddings and extended topic information. Following this, two fusion strategies of weighted word embeddings and extended topic information are designed: static linear fusion and dynamic fusion. This method can highlight important semantic information, flexibly fuse topic information, and improve the capabilities of short text representation. We use classification and prediction tasks to verify the effectiveness of the method. The testing results show that the method is valid.

## 1. Introduction

With the rise and the widespread use of social media platforms, huge amounts of text data are generated every day. The text usually contains a lot of information, such as emotions and positions. However, text is unstructured data, which leads to time-consuming and laborious manual analysis. Figuring out how to represent unstructured text as a distributed vector that can be easily recognized by a computer is very important [1]. Therefore, text representation has become more and more important in natural language processing (NLP). A good representation method should fully learn the grammatical and semantic information in natural language and lay a solid foundation for downstream tasks, such as text classification and sentiment analysis [2]. In addition, training deep learning models of text representation through labeled datasets usually requires a lot of manual work [3]. Therefore, we will focus on the unsupervised learning of short text representation, which includes abstracts, instant messaging, social reviews, etc. (the short text studied in this paper mainly refers to the text with a length of no more than 512 words).

In recent years, the distributed word representation model based on deep neural networks has developed rapidly [4]. Although these models can use the semantic relevance of the distributed words and their representation, with the approximate addition and subtraction characteristics in semantic space, they lose part of the global information, and this affects the representation accuracy to some extent. The topic model can represent the topic information contained in the text, which can represent the global information of the text. Jiang et al. (2020) have proved that introducing global information into a distributed semantic representation vector can capture semantic information better [2]. According to this idea, we propose a short text representation method, which is based on weighted word embeddings (WWE) and extended topic information (ETI).

The main contributions of our work can be summarized as follows:

1. This paper designs a short text representation method based on weighted word embeddings and extended topic information. In order to integrate the global topic information implied into the text semantic vector space, two fusion strategies are designed to fuse the semantic representation vector and the topic vector.
2. We propose a weighted word embedding method, and this method uses Word2Vec for word embedding and *TF-IWF* [5] to measure the importance of words.
3. We propose a word sequence extension method based on a sliding window without changing the word order and semantics of the original text. The method enriches the topic information of short text and solves the problem of the sparse topic vector of short text to a certain extent.
4. This paper uses two classification task datasets to evaluate the performance of the proposed method, and compares it with the current representative text representation methods. The experimental results show that the WWE and ETI methods are better than the baseline method; the fusion method with text length as the basic variable is relatively reasonable. For text classification tasks, the static fusion method and dynamic fusion method have better performance than traditional methods.

The rest of the paper is organized as follows. In Section 2, some related work is reviewed, including the topic models and deep learning models. Section 3 introduces WWE and ETI, which we propose for short text representation and their fusion methods. In Section 4, the performance is evaluated with experiments on popular datasets. Finally, the conclusion and future work are given in Section 5.

## 2. Related Work

In this section, we briefly describe the related work on the following two themes: text representation based on topic models and deep learning models.

### 2.1. Topic Models

The topic model is the earliest text analysis tool and most popular language model [6]. It is an effective unsupervised tool, which can reveal the latent topic information according to the co-occurrence of information in the text corpus. At present, it has penetrated into multiple fields, such as topic extraction, text clustering, text classification, social network relationship analysis, and sentiment analysis. The topic model, in the usual sense, refers to probabilistic latent semantic analysis (PLSA) [7], the LDA [8], and various extensions [9]. The LDA introduces conjugates prior to Dirichlet distribution based on PLSA. It believes that each document is a mixture of multiple topics, each topic is a probability distribution of a set of words, and each word in the document is composed of a fixed theme generated. The LDA solves the connection between polynomial parameters and variables. However, the traditional topic model based on LDA mainly relies on the co-occurrence relationship of terms in long documents to establish a model. Moreover, the LDA model has a relatively sensitive text selectivity problem, which makes its performance on short text easily limited. Masada et al. (2015) and Quan et al. (2016), through analysis, find that the short text itself is short in length, and each word appears only once in the document, which causes the severe data sparsity problem [10,11]. Mehrotra et al. (2013) aggregated multiple tweets into one pseudo-text based on tags [12]. In another scheme, Qiang et al. (2017) aggregated short text into pseudo-text by fusing the semantic knowledge embedded in the words, and then used a Markov random field regularization model to infer topics from long pseudo-text, in which the clustering method faced the same problem of very limited word co-occurrence information [13]. Although the NMF model proposed by Shi et al. (2018) makes use of the relevance of word context semantics, it does not really solve the problem of sparse topic information [14]. To some extent, the above methods can complete the task of identifying the specific topic, but more generally, it is necessary to find the topic information contained in each short text, and these short texts hardly provide any such contextual information.

In order to solve the above problems, some excellent topic models have emerged in recent years, mainly including a mixture of unigrams (Mix-gram), the word network topic model (WNTM) [15], and the biterm topic model (BTM) [16]. Mix-gram assumes that each document contains only one topic, which is not quite true. The word co-occurrence model constructed by the other two models may lead to a sharp increase in the number of word pairs or the loss of potential coherent word pairs. In any case, these three models give us great inspiration; in order to improve the quality of short text topic modeling, we will propose a new method of short text topic modeling based on text extension.

### 2.2. Deep Learning Models

As mentioned in the introduction, text representation is the keystone for text mining and NLP tasks. According to the granularity of text representation, the relevant methods can be divided into word-level, sentence-level, and document-level representation. The distributed word representation method, also known as the word embedding method, is currently recognized as the most effective tool for text representation. Most text representations are improved on the basis of distributed word embedding. Embedding was originally a proper term in the field of mathematics, which means that object A is mapped or embedded in B. When applied to NLP, word embedding also refers to a mapping relationship, which refers to mapping a word or phrase in the word table to a vector composed of real numbers. In 1986, the word vector model was first proposed by Hinton et al. [17]. At that time, the idea of a distributed representation of words was proposed in the paper, but it has not been taken seriously until recent years. The main idea of a word vector is to link every one-hot word to a lower-dimensional vector through training, so as to solve the two shortcomings of one-hot word representation (being unable to express the relationship between words, and dimensional disaster). In 2003, Bengio (2003) proposed a language model consisting of a feedforward neural network that could be generated using the N-gram method [18]. The initial word vector training process is accompanied by the generation of the language model. With the rise of neural networks, a large number of neural network models suitable for natural language have been proposed. In 2013, Mikolov (2013), on the basis of the neural network language model (NNLM) and the recurrent neural network language model (RNNLM), proposed the famous Word2Vec. Pennington et al. (2014), based on global word sharing, proposed the Glove [19]. Due to the rapid development of computer hardware, a large number of language models based on deep learning have been proposed, such as ELMo (Embeddings from Language Modeling) [20], GPT (Generative Pre-training), and BERT (Bidirectional Transformer for Language Understanding) [21]. Although ELMo, GPT, and BERT can dynamically adjust the word semantics according to the context information to achieve more accurate representation, due to a large number of parameters for this kind of model, it requires significant computer hardware resources and has poor real-time performance. Therefore, in order to better adapt to the representation of a large number of short text data, this paper takes Word2Vec as the basic word embedding model and proposes a new word embedding method to represent short text by identifying important words and increasing the weight of important words.

## 3. Architecture and Methods

This section introduces our proposed method of short text representation, including the architecture of short text representation, WWE and ETI, and the fusion methods of WWE and ETI. Table 1 lists a description of the notation that appears in this section.

### 3.1. Architecture for Short Text Representation

**Figure 1:**
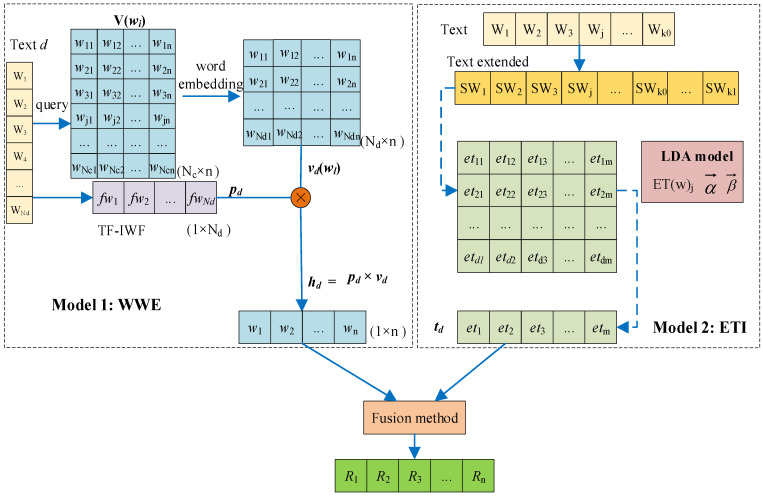
Architecture of short text representation.

The architecture of the short text representation mainly includes the weighted word embeddings model, WWE (based on Word2Vec and *TF-IWF*), and the extended topic information method, ETI (based on word sequence extension and the LDA topic model). The architecture of short text representation is shown in Figure 1.

The left of Figure 1 describes the process of obtaining the weighted word embeddings.

- According to the corpus *C*, generate a dictionary *V* of common words.
- Based on the CBOW [22] model, the corpus *C* is used to train the CBOW model, and the word embeddings dictionary $V(w_{i})\in R^{N_{C}\times n}$ of *V* can be obtained.
- The word vector distribution $v_{d}(w_{l})\in R^{N_{d}\times n}$ of short text *d* is obtained by querying the word embeddings dictionary $V(w_{i})\in R^{N_{C}\times n}$.
- Calculate the *TF-IWF* of the short text *d* to obtain the weight of each word in the short text *d*, remarked as $p_{d}(fw_{j})\in R^{1\times n}$.
- Generate WWE $h_{d}\in R^{1\times n}$ for short text representation through the multiplication of vectors $p_{d}(fw_{j})$ and $v_{d}\left(w_{i}\right)$.
- The right of Figure 1 describes the process of obtaining the extended topics.
- The word sequence $List_{d}$ of each short text in corpus *C* is extended according to the sliding window, and the extended word sequence $List_{d}^{\prime }$ of each short text is obtained. At the same time, we also obtain the corpus $C^{\prime }$.
- The LDA topic model is used to train the corpus.
- With each short text $List_{d}^{\prime }$ as the input of the trained LDA model, the extended topic vector $t_{d}\left({et}_{i}\right)\in R^{1\times n}$ of each short text is obtained.
- Finally, the short text representation $R_{d}$ is obtained by fusing $h_{d}$ and $t_{d}(et_{i})$.

### 3.2. WWE Model

**Figure 2:**
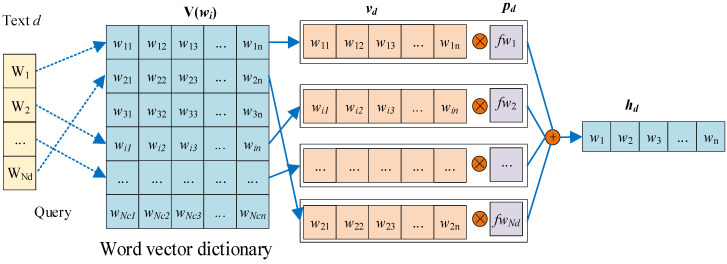
Model of the WWE.

We propose a WWE model based on *TF-IWF* and CBOW. The model of WWE is shown in Figure 2.

Although many scholars use *TF-IDF* to weight the words in the vector, which greatly improves the document representation based on a static word vector, *IDF* reflects the importance of words and the distribution of feature words based on the quotient of the total number of documents divided by the number of documents with specific words. When a word appears in more than one document, the smaller the quotient is, the less important the word is, and the more inaccurately it is reflected in the specific corpus environment. Because, in a domain, a word appears in different documents many times, this just reflects the fact that the word is more important. Different from *IDF*, *IWF* reduces the influence of similar texts on word weight in the corpus, and more accurately expresses the importance of words in the documents to be checked. For example, when a word appears in multiple documents, but the total word frequency of the word is relatively small, the IWF calculation result will be larger, indicating that the word is more important, which is close to the fact. We use *TF-IWF* to weight the words in the vector for short text.

*TF-IDF* is a commonly used feature weighting technology in information retrieval and text mining, and is often used in text topic extraction and word segmentation weighting. *TF-IDF* is a completely statistical method. Its core idea is to assume that the importance of a word is proportional to the proportion of its appearance in a certain document, and inversely proportional to its proportion of appearances in other documents. It is defined as Equation (1):

(1) $$TF-IDF=tf_{ij}\times idf_{i}=\frac{n_{i,j}}{\sum_{k}n_{k,j}}\times \log\frac{\left|C\right|}{\left|\{j:t_{i}\in d_{j}\}\right|}$$

*TF* means word frequency, that is, the number of times a word appears in the document. This may be positively related to the length of the document. Therefore, the word frequency needs to be normalized, usually by dividing the number of times it appears by the total number of words in the document:

(2) $$tf_{ij}=\frac{n_{i,j}}{\sum_{k}n_{k,j}}$$

In Formula (2), the numerator $n_{i,j}$ denotes the frequency of the word $t_{i}$ in document *j*, and the denominator $\sum_{k}n_{k,j}$ denotes the sum of all vocabularies in the document *j*.

*IDF* represents the inverse document frequency, defined as the total number of documents divided by the number of documents containing a given word. In Equation (3), |*C*| denotes the total number of documents in corpus *C*, and the denominator $\left|\left\{j:t_{i}\in d_{j}\right\}\right|$ represents the number of documents in corpus *C* that contain the word $t_{i}$ in the document *j*. In applications, in order to avoid the denominator being 0, the denominator is generally given as $\left|1+\left\{j:t_{i}\in d_{j}\right\}\right|$.

(3) $$idf_{i}=\log\frac{\left|C\right|}{\left|\{j:t_{i}\in d_{j}\}\right|}$$

In essence, *IDF* is a weighting method that tries to suppress noise. It assumes that less frequent words are more important and more frequent words are less important. This is not entirely correct for most text information. The keywords extracted by *IDF* cannot effectively reflect the importance of words and the distribution of characteristic words if the model is then unable to complete the function of weight adjustment well. Especially in similar corpora, this method has flaws, and often some keywords of the same text are covered.

To solve the shortcomings of *IDF* with short text and similar corpora, this paper adopts *TF-IWF* to weight the word embedding in each text. TF is consistent with the definition in *TF-IDF*, and IWF is defined as Equation (4):

(4) $$iwf_{i}=\log\frac{\sum_{i=1}^{m}nt_{i}}{nt_{i}}$$

In Equation (4), the numerator $\overset{m}{\underset{i=1}{\sum }}nt_{i}$ represents the sum of the frequencies of all words in the corpus, and the denominator $nt_{i}$ represents the total frequency of the word $t_{i}$ in the corpus. Therefore, *TF-IWF* is defined by Equation (5):

(5) $$TF-IWF_{i,j}=tf_{ij}\times iwf_{i}=\frac{n_{i,j}}{\sum_{k}n_{k,j}}\times \log\frac{\sum_{i=1}^{m}nt_{i}}{nt_{i}}$$

According to Formula (5), calculate the $p_{ij}$ based on *TF-IWF* for each text in the corpus, denoted by $p_{ij}=(fw_{i1},fw_{i2},fw_{i3},\ldots ,fw_{ij})$, where *i* represents the text number, and *j* represents the *j*th word in the text.

Suppose that corpus *C* forms a vocabulary *V*, and each word in the vocabulary *V* is encoded by the one-hot method. According to the idea of the CBOW model, each word (one-hot encoding) in vocabulary *V* can be mapped into a low-latitude dense word vector through training, which is denoted as matrix $V(w_{i})$. Therefore, we can obtain the word vector $v(w_{i})$ representation of short text by querying $V(w_{i})$, and finally generate WWE $h_{d}$ for each short text representation through the multiplication cross of vectors $p(fw_{j})$ and $v\left(w_{i}\right)$.

(6) $$h_{d}=dot(p(fw_{j}),v(w_{i}))$$

### 3.3. Extended Topic Information

The sparsity of content in short text brings new challenges to topic modeling [23]. Conventional topic models assume that a document is a mixture of topics, where a topic is seen as conveying a certain semantic meaning through a set of correlated words. Then, they utilize statistical techniques to learn the topic component and mixing coefficient of each document. In essence, the conventional topic model reveals the topics in the corpus by implicitly capturing document-level word co-occurrence patterns. Therefore, applying these models directly to short text will suffer from severe data sparsity problems. On the one hand, the frequency of occurrence of words in a single short text is less than that of a long text, so it is difficult to infer which words in each document are more relevant. On the other hand, because the short text contains sparse words, it cannot express rich topic information by means of global co-occurrence.

The purpose of this paper is to enrich the semantic information of short text through topic information, while the LDA model exploits word co-occurrence patterns to reveal the latent semantic structure of a corpus in an implicit way by modeling the generation of words in each document. In order to solve the problem of sparse and weakened short text topic representation, we learn from the idea of BTM [23] for extending the topic information without changing the original document’s words. With this idea, we use a novel method to extend the latent topic components in short text by directly extending word sequences. The detailed process of generating topic information with the LDA model (https://doi.org/10.1007/s44196-021-00055-4, accessed on 26 January 2022) can be found in reference [24].

Before we detail the method of word expansion, we first introduce the “biterm” in the BTM model. Biterm denotes an unordered word pair co-occurring in a short context. In such a case, any two distinct words in a document construct a biterm. For example, a document with three distinct words will generate three biterms:

$$\begin{array}{c} List_{m}=\langle (w_{m1}),(w_{m2}),\left(w_{m3}\right)\rangle \\ ⇓\\ List_{m}^{\prime }=\langle (w_{m1},w_{m2}),(w_{m1},w_{m3}),\left(w_{m2},w_{m3}\right)\rangle \end{array}$$

In $List_{m}^{\prime }$, the biterm is unordered, and the whole corpus turns into a biterm set. Although this method greatly enriches the topic information, this topic information is not suitable for representing the semantic information of short text. This method does not consider the word order of the source document (which may cause confusion with the semantic information of the short text) and does not retain the original words (losing part of the original subject information). Therefore, we adopt the following method for adaptive transformation, and other parts of the ETI are consistent with traditional LDA.

$$List_{m}^{\prime }=\langle (w_{m1}),(w_{m1}w_{m2}),(w_{m2})(w_{m2}w_{m3}),\ldots \rangle$$

After the extension, we can use the LDA model-generated topic representation. The theory above can greatly improve the weakness of the LDA model with short text. Therefore, we can obtain topic information for each text $t_{d}$.

(7) $$t_{d}=LDA([et_{1},et_{2},\ldots et_{i}])\in R^{n}$$

### 3.4. Fusion Method

Through the above steps, we can obtain a distributed representation of each short text $h_{d}$ and $t_{d}$. Although they represent semantic information and topic information to a certain extent, their proportions should be different for each short text. Therefore, we propose two fusion strategies:

Static linear fusion: This is achieved by assigning a static parameter to $h_{d}$ and $t_{d}$ to adjust semantic information and topic information, as shown in Equation (8). The λ stands for the weight, and the empirical value range is 0 to 1.

(8) $$R_{d}=\lambda \cdot h_{d}+(1-\lambda )\cdot t_{d}$$

Dynamic fusion: The WWE gives different weights to each word vector and obtains the distributed representation of short text by accumulating the weighted word vectors. Due to the different lengths of the texts, this accumulation method will lead to differences between semantic representation and topic representation. The more words there are in the text, the greater the noise, and the more easily the semantic information on $h_{d}$ is obscured. On the contrary, $t_{d}$ has more topic information. Therefore, we propose a dynamic fusion model, as shown in Equation (9):

(9) $$R_{d}=(1-\frac{Len}{\delta })h_{d}+\frac{Len}{\delta }\cdot t_{d}$$

The research object of this paper is short text with less than 512 words, such as paper abstracts, instant messages, and website reviews. For short texts that are more than 512 words in length, we truncate them and keep only the first 512 words. In Equation (9), *Len* denotes the number of effective words contained in short text, and so its maximum value is 512 in this paper; *δ* is a hyperparameter used to adjust the performance of the formula, and it can be any value greater than max (*Len*), and max (*Len*) = 512 in this paper. In addition, by observing Equation (9), we can find that *δ* can be used to adjust the margin of $h_{d}$ and $t_{d}$; that is, the smaller the *δ* is, the larger the adjustment is. Otherwise, the smaller the adjustment is.

## 4. Experiments and Discussion

In this section, we use classification tasks to evaluate our proposed short text representation models, WWE and ETI, and their performance after fusion. The text representation models we proposed are unsupervised, but in order to prove their validity, experiments are carried out using labeled datasets from IMDB and 20 Newsgroups. In order to focus on the text representation method and evaluate the performance of our proposed model, the experimental method of single-label classification is used to compare the text representation performance of multiple classifiers. The experiment consists of four parts:

- Dataset analysis and preprocessing;
- Measure of performance;
- Baseline models;
- Experiments and results analysis.

### 4.1. Dataset Analysis and Preprocessing

We use the following two text classification datasets: Internet Movie Database (IMDB), and 20 Newsgroups.

The IMDB dataset contains 50,000 highly polarized comments from the Internet Movie Database. The dataset is divided into 25,000 comments for training and 25,000 comments for testing. Both the training set and the testing set contain 50% positive comments and 50% negative comments.

The 20 Newsgroups dataset is one of the international standard datasets for text classification, text mining, and information retrieval. There are three versions of the dataset. This paper uses the latest version. The dataset collects 18,828 non-duplicate news documents, which are evenly divided into 20 news groups with different topics.

Table 2 shows the length distribution of the original text in IMDB and 20 Newsgroups.

In Table 2, *Len* represents the length of the text, while *QTY* and *PCT* represent the number and percentage of the corresponding length of the text in the training set.

- In order to better represent the text by semantic vector and topic vector, we adopt the following preprocessing strategies:
- Remove invalid symbol: Remove punctuation and special symbols in the text, e.g., # @ ? < *.
- Digital normalization: The Arabic numerals are replaced by the English word “number”. Because the specific number symbols have no practical significance, and the Arabic numerals are infinite, it is difficult to accurately represent the text features. Replacing the specific number with “number” can retain the semantics of the original text to a certain extent.
- Convert case: Convert all uppercase English characters to lowercase.
- Lemmatization: Lemmatization means to change a word into its original form, such as “drove” to “drive” and “driving” to “drive”.

### 4.2. Measure of Performance

**Figure 3:**
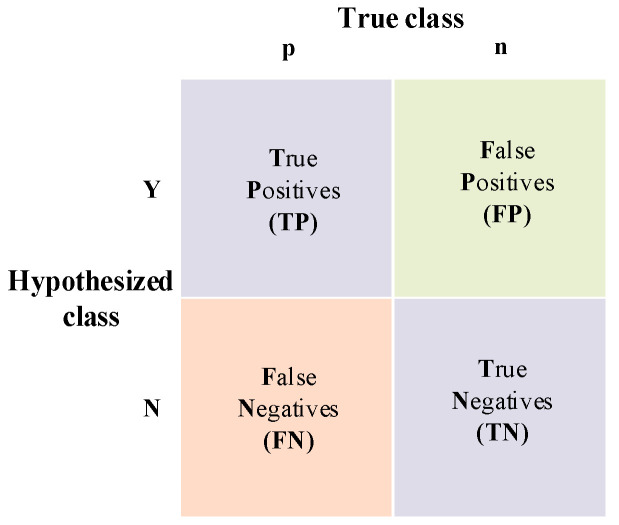
Confusion matrix.

This paper uses accuracy and the F1-score to measure the effect of classification. Accuracy is a common metric in deep learning research, and F1 is a common criterion for classification tasks. Suppose the confusion matrix is shown in Figure 3.

The calculation formulas for *Accuracy*, *Precision*, and *Recall* are as follows:

(10) $$Accuracy=\frac{TP+TN}{TP+TN+FP+FN}$$

(11) $$Precision=\frac{TP}{TP+FP}$$

(12) $$Recall=\frac{TP}{TP+FN}$$

The *F*1*__score_* can take precision and recall into account:

(13) $$F1_{\_score}=\frac{2*Precision*Recall}{Precision+Recall}$$

This paper uses both accuracy and *F*1*__score_* to measure classification results.

### 4.3. Comparison Method and Parameter Setting

This paper proposes a short text representation method based on the weighted word embedding vector and extended topic information, which consists of three parts: short text semantic feature representation based on WWE and extended topic feature representation based on ETI, and their fusion strategy. Therefore, the selected baseline models also use the class method or model, and select the k-nearest neighbor (KNN) classifier and support vector machine (SVM) classifier, with classification tasks as the basis to verify the effectiveness of the proposed short text representation method.

#### 4.3.1. Comparison Method

- LDA: This method constructs the document-level theme vector and the word-level topic vector through the word global co-occurrence relationship. This paper selects the document-level theme vector.
- W2V: This method directly accumulates the word vectors of each word to represent the short text, and the word embedding vector is obtained by the Word2Vec [8] pre-training model. The method is realized by local context features.
- GLV [17]: This method directly accumulates the word vectors of each word to represent the short text, and the word embedding vector is obtained using the Glove pre-training model. This method is based on global word vector sharing.
- FPW [3]: This method is based on a topic vector and word embedding vector; that is, the short text is represented by accumulating the topic vector of the short text and the word vector of each word.

#### 4.3.2. Our Method

It can be seen from Section 3 that the short text representation method proposed in this paper consists of three parts: WWE, ETI, and the fusion method. Therefore, the methods of participating in the comparative experiment can be summarized as WWE, ETI, and the static fusion method (SFM) and the dynamic fusion method (DFM).

#### 4.3.3. Parameter Setting

In order to facilitate the efficient comparison between different methods, the dimensionality of Word2Vec, Glove word vector, and topic vector based on LDA used in the experimental part is unified as 100d.

For the Glove word vector, this paper selects the pre-trained Glove100d of the StanfordNLP (https://github.com/stanfordnlp/GloVe, accessed on 26 January 2022) as the word embedding vector. That is, the word embedding vector dimensionality of each word is 100d.

- For Word2Vec, this paper uses the Word2Vec model of genism library for word vector training. The parameters of the Word2Vec model are: *size* = 100 (word vector dimensionality), *window* = 5 (word vector context maximum distance), *sg* = 0 (CBOW model), *hs* = 0 (negative sampling), *negative* = 5 (number of negative sampling). For other parameters, we choose the default values.
- For the LDA topic model, we set the number of topics k=100, and the hyperparameters α=0.5, β=0.01.
- For the method of obtaining the ETI topic vector, LDA is still used in the topic model, and the parameters remain unchanged. For the short text word sequence expansion, we set the sliding window to 2; that is, two consecutive words can form a new word. In this way, the length of the short text is basically expanded to two times the original.
- We choose the SVM and KNN model of the scikit-learn library as the classifier. For the kernel function of the SVM classifier, we choose sigmoid as the kernel function, and the parameters of the kernel function adopt the default values of the model. For the KNN classifier, the K value is set according to the category of our dataset, with weights = ‘uniform’ (distance weight is not considered).

### 4.4. Experiments and Results Analysis

This section shows the experimental results and analysis on the IMDB and 20 Newsgroups training datasets, in which there are 25,000 texts in IMDB and 11,314 texts in 20 Newsgroups. In addition, in order to reduce the overfitting, the k-fold cross-validation method (k = 5) was used in the experiment. Dataset preprocessing, the evaluation metric, the comparison method, and parameter settings are set according to the first three sections of this article. The experiment is divided into four parts:

First, we compare and analyze the influence of different text lengths on the text representation method (Table 3 and Table 4). Secondly, according to Formula (8), we compare and verify the static fusion strategy by choosing different λ (Table 5 and Table 6). The third is where, according to Formula (9), we compare and verify the dynamic fusion strategy by choosing different *δ* (Table 7 and Table 8). Fourth, we compare the short text representation methods, such as WWE, ETI, SFM, and DFM, with the baseline methods (Table 9 and Table 10).

#### 4.4.1. The Effect of Length on Text Representation

Table 3 and Table 4 show the performance of different text representation methods on the classification task under two classifiers and with multiple text length partition strategies. We can find that with the increase in the text length, W2V, GLV, and our proposed WWE text representation methods lead to a decline in the scores for the performance of classification tasks, whether on the IMDB dataset or 20 newsgroups dataset. On the contrary, LDA and ETI improve the performance of the classification tasks with the increase in text length. It also verifies that the proposed text length has a certain influence on the text representation methods, such as the word vector direct superposition method and LDA topic vector method. In addition, we can also find that our WWE method is superior to the method of directly using Word2Vec or Glove word vector superposition. Compared with the LDA method, the ETI method has better performance, especially when the text length is short.

In addition, although different classifiers have an impact on the experimental results, this paper focuses on the impact of text representation and its fusion strategy. Therefore, in order to facilitate the comparison of experimental results, some experiments only use an SVM classifier.

#### 4.4.2. Static Fusion Strategy Analysis

Table 5 and Table 6 show the performance of the text representation method in the classification task with different text length partition strategies and different static fusion strategies (λ takes different values). We can find that, whether on the IMDB dataset or 20 Newsgroups dataset, with the increase in text length, when the text classification task achieves better results, the value of λ decreases. For example, on the IMDB dataset, when the text division strategy is *Len* < 100, λ = 0.9, and the best classification result can be obtained; when *Len* ≥ 400 and λ=0.3, the best classification result can be obtained. The 20 Newsgroups dataset has the same conclusion. From Table 5, it can be found that under the static fusion strategy, when λ=0.5, the best classification effect can be achieved for the entire IMDB dataset. It can be found from Table 6 that under the static fusion strategy, when λ=0.7, the best classification effect can be achieved for the entire 20 Newsgroups, which is related to the characteristics of the dataset itself and the length distribution. At the same time, this also preliminarily verifies the rationality of the text length as the basic variable in our proposed fusion method (Equations (8) and (9)).

In addition, by observing and comparing Table 3, Table 4, Table 5 and Table 6, we can find that on the IMDB and 20 Newsgroups datasets, the text classification effect under the static fusion strategy is generally better than the single text representation method, which also confirms that the topic vector superimposing the word vectors can improve the accuracy of text feature representation.

#### 4.4.3. Dynamic Fusion Strategy Analysis

Table 7 and Table 8 show the performance of the text representation method on the classification task under different text length divisions and different dynamic fusion strategies (different values of δ). Since our short text representation object is where the number of words contained in the text is less than 512, δ should be greater than 512 according to Equation (9). In addition, in order to provide a concise experimental scheme and a clear comparison of experimental results, we set the value of δ to 600, 700, 800, 900, and 1000. We can find that whether it is on the IMDB dataset or the 20 Newsgroups dataset, the text classification results are more stable and perform better than the static fusion strategy. For the IDMB dataset, when the value of δ is around 800, generally better results can be obtained; on the 20 Newsgroups dataset, when the value of δ is around 700, generally better results can be obtained. This is mainly related to the length distribution of the dataset itself. By observing Table 2, it can be found that the text length of the IMDB dataset is relatively concentrated, and the proportion of the text length between 100 and 200 is 46.7%, while the 20 Newsgroups dataset has a relatively uniform proportion of text lengths below 300.

#### 4.4.4. Comparison and Analysis

Table 9 and Table 10 show the performance of different text representation methods with the classification tasks. For horizontal comparison, we use all training sets (without length division) for the experiments. The text representation method we proposed is better than the baseline methods in the text classification tasks, especially the DFM method, which achieves the best performance in most cases. In addition, by observing the experimental results, it can be concluded that both SFM and DFM are superior to WWE and ETI in terms of text feature representation, which also proves that integrating topic vectors into the word vector space can improve text representation and the performance of downstream classification tasks.

## 5. Conclusions and Future Work

In this work, we improved two models, weight word embedding and extended topic information, and proposed two fusion methods based on these two models. All of them aim to address issues of fuzzy and high-dimensional sparse semantic feature extraction with short text representation. In addition, the WWE model has better representation capabilities for shorter text, while ETI has better representation capabilities for relatively long text. In order to make the model performance stable, we use these two models to design the static linear fusion and dynamic fusion methods, respectively. The short text representation adopted in our methods is able to capture key and meaningful semantic information with integrated topic information. They are not aimed at specific tasks and have universal adaptability. Next, we will study some new word embedding methods, feature weighting techniques, and more efficient topic information extension methods to obtain better text representation models. At the same time, we will also try task-based end-to-end text representation methods to apply in key areas of concern.

## Figures and Tables

**Table 1:** Notation description in this section.

Notation	Description
$N_{C}$	Number of words in corpus *C*
$N_{d}$	Number of words in short text *d*
$N_{d}^{\prime }$	Number of words in extended short text *d*
n	*n* denotes the the dimensionality of a vector
$p_{d}(fw_{j})\in R^{1\times n}$	*TF-IWF* vectors of short text *d*
$V(w_{i})\in R^{N_{C}\times n}$	Word vector dictionary of corpus *C*
$v_{d}(w_{l})\in R^{N_{d}\times n}$	Word embeddings of short text *d*
$h_{d}$	WWE of short text *d*
$t_{d}(et_{i})\in R^{1\times n}$	Topic vectors of extended short text *d*, where *n* denotes the number of topics
$w_{m,n}$	The *n*th word of document *m*
$z_{m,n}$	The *n*th topic related to document *m*
$\varphi_{k},k\in [1,K]$	Word distribution of topic, where *K* denotes the total number of potential topics
$\theta_{m},m\in [1,M]$	Topic distribution of the *m*th document, where *M* denotes the total number of documents
α,β	Prior hyperparameter, generally set as α=50/K;β=0.01
$List_{d}$	Word list of short text *d*
$List_{d}^{\prime }$	Word list of short text *d* after word extended
$R_{d}$	Fusion of WWE and ETI for representing short text *d*
dot(A,B)	Denotes multiplication cross of matrix *A* and matrix *B.*

**Table 2:** The length distribution of original text in IMDB and 20 Newsgroups.

Datasets	*Len* < 100		100 ≤ *Len* < 200		200 ≤ *Len* < 300		300 ≤ *Len* < 400		*Len* ≥ 400	
	*QTY*	*PCT*	*QTY*	*PCT*	*QTY*	*PCT*	*QTY*	*PCT*	*QTY*	*PCT*
IMDB	2950	11.8%	11680	46.7%	4665	18.7%	2402	9.6%	3303	13.2%
20 Newsgroups	2027	17.9%	4147	36.7%	2301	20.3%	1124	9.9%	1715	15.2%

**Table 3:** Results of the influence of different text lengths on different representation models (IMDB).

Model	Classifier	*Len <* 100		100 ≤ *Len <* 200		200 ≤ *Len <* 300		300 ≤ *Len <* 400		*Len* ≥ 400	
		*Acc*	*F*1	*Acc*	*F*1	*Acc*	*F*1	*Acc*	*F*1	*Acc*	*F*1
W2V	KNN	0.9461	0.9442	0.9485	0.9454	0.9432	0.9411	0.9453	0.9421	0.9405	0.9399
	SVM	0.9525	0.9501	0.9392	0.9388	0.9475	0.9444	0.9436	0.9417	0.9423	0.9403
GLV	KNN	0.9510	0.9493	0.9441	0.9413	0.9442	0.9426	0.9408	0.9383	0.9284	0.9265
	SVM	0.9491	0.9475	0.9504	0.9487	0.9452	0.9430	0.9393	0.9364	0.9275	0.9255
WWE	KNN	0.9531	0.9525	0.9557	0.9532	0.9555	0.9543	0.9504	0.9487	0.9491	0.9472
	SVM	0.9504	0.9483	0.9545	0.9504	0.9534	0.9513	0.9537	0.9501	0.9463	0.9404
LDA	KNN	0.8602	0.8565	0.8624	0.8607	0.8653	0.8631	0.8766	0.8744	0.8732	0.8713
	SVM	0.8581	0.8572	0.8584	0.8573	0.8651	0.8647	0.8772	0.8754	0.8781	0.8745
ETI	KNN	0.8774	0.8752	0.8806	0.8787	0.8822	0.8815	0.8876	0.8757	0.8821	0.8808
	SVM	0.8797	0.8765	0.8814	0.8796	0.8798	0.8761	0.8856	0.8834	0.8846	0.8822

**Table 4:** Results of the influence of different text lengths on different representation models (20 Newsgroups).

Model	Classifier	*Len <* 100		100 ≤ *Len <* 200		200 ≤ *Len <* 300		300 ≤ *Len <* 400		*Len* ≥ 400	
		*Acc*	*F*1	*Acc*	*F*1	*Acc*	*F*1	*Acc*	*F*1	*Acc*	*F*1
W2V	KNN	0.7313	0.7291	0.7354	0.7332	0.7154	0.7133	0.7192	0.7181	0.7119	0.7092
	SVM	0.7182	0.7164	0.7091	0.7075	0.7112	0.7102	0.7063	0.7055	0.6892	0.6872
GLV	KNN	0.7285	0.7257	0.7212	0.7196	0.7223	0.7195	0.7156	0.7132	0.7083	0.7064
	SVM	0.7196	0.7183	0.7154	0.7142	0.7116	0.7109	0.7081	0.7067	0.7054	0.7035
WWE	KNN	0.7398	0.7374	0.7473	0.7454	0.7515	0.7494	0.7364	0.7345	0.7281	0.7262
	SVM	0.7297	0.7275	0.7385	0.7363	0.7354	0.7337	0.7319	0.7301	0.7252	0.7231
LDA	KNN	0.6542	0.6521	0.6592	0.6571	0.6662	0.6635	0.6692	0.6672	0.6675	0.6654
	SVM	0.6616	0.6596	0.6581	0.6562	0.6691	0.6672	0.7013	0.7015	0.7133	0.7119
ETI	KNN	0.6827	0.6794	0.6876	0.6851	0.6915	0.6891	0.6832	0.6816	0.6881	0.6862
	SVM	0.6929	0.6903	0.6954	0.6939	0.6909	0.6886	0.6997	0.6974	0.6945	0.6935

**Table 5:** Comparison of SFM results with different λ by SVM classifier (IMDB dataset).

Text Partition Strategy	λ = 0.1		λ = 0.3		λ = 0.5		λ = 0.7		λ = 0.9	
	*Acc*	*F*1	*Acc*	*F*1	*Acc*	*F*1	*Acc*	*F*1	*Acc*	*F*1
*Len* < 100	0.9416	0.9394	0.9452	0.9434	0.9581	0.9563	0.9577	0.9556	0.9634	0.9616
100 ≤ *Len* < 200	0.9553	0.9535	0.9643	0.9622	0.9712	0.9692	0.9704	0.9681	0.9692	0.9672
200 ≤ *Len* < 300	0.9742	0.9721	0.9715	0.9705	0.9784	0.9765	0.9752	0.9731	0.9771	0.9753
300 ≤ *Len* < 400	0.9735	0.9724	0.9774	0.9752	0.9705	0.9682	0.9665	0.9643	0.9695	0.9672
*Len* ≥ 400	0.9687	0.9662	0.9753	0.9732	0.9612	0.9593	0.9531	0.9515	0.9572	0.9551
All	0.9551	0.9538	0.9581	0.9563	0.9661	0.9641	0.9574	0.9552	0.9653	0.9632

**Table 6:** Comparison of SFM results with different λ by SVM classifier (20 Newsgroups dataset).

Text Partition Strategy	λ = 0.1		λ = 0.3		λ = 0.5		λ = 0.7		λ = 0.9	
	*Acc*	*F*1	*Acc*	*F*1	*Acc*	*F*1	*Acc*	*F*1	*Acc*	*F*1
*Len* < 100	0.7295	0.7271	0.7162	0.7154	0.7381	0.7365	0.7454	0.7432	0.7415	0.7391
100 ≤ *Len* < 200	0.7352	0.7332	0.7325	0.7302	0.7493	0.7472	0.7516	0.7492	0.7473	0.7463
200 ≤ *Len* < 300	0.7514	0.7492	0.7653	0.7635	0.7685	0.7663	0.7612	0.7591	0.7581	0.7562
300 ≤ *Len* < 400	0.7693	0.7674	0.7781	0.7762	0.7743	0.7754	0.7735	0.7715	0.7642	0.7625
*Len* ≥ 400	0.7442	0.7425	0.7472	0.7453	0.7392	0.7372	0.7364	0.7345	0.7316	0.7291
All	0.7415	0.7403	0.7393	0.7372	0.7454	0.7432	0.7553	0.7532	0.7481	0.7465

**Table 7:** Comparison of DFM results with different δ by SVM classifier (IMDB dataset).

Text Partition Strategy	δ = 600		δ = 700		δ = 800		δ = 900		δ = 1000	
	*Acc*	*F*1	*Acc*	*F*1	*Acc*	*F*1	*Acc*	*F*1	*Acc*	*F*1
*Len* < 100	0.9441	0.9423	0.9536	0.9516	0.9591	0.9572	0.9675	0.9651	0.9624	0.9604
100 ≤ *Len* < 200	0.9716	0.9692	0.9773	0.9754	0.9775	0.9754	0.9846	0.9824	0.9775	0.9762
200 ≤ *Len* < 300	0.9792	0.9775	0.9832	0.9815	0.9733	0.9713	0.9783	0.9767	0.9692	0.9674
300 ≤ *Len* < 400	0.9713	0.9694	0.9784	0.9762	0.9768	0.9748	0.9802	0.9796	0.9851	0.9831
*Len* ≥ 400	0.9754	0.9747	0.9731	0.9713	0.9854	0.9847	0.9819	0.9798	0.9803	0.9792
All	0.9742	0.9736	0.9709	0.9694	0.9776	0.9765	0.9737	0.9713	0.9752	0.9746

**Table 8:** Comparison of DFM results with different δ by SVM classifier (20 Newsgroups dataset).

Text Partition Strategy	δ = 600		δ = 700		δ = 800		δ = 900		δ = 1000	
	*Acc*	*F*1	*Acc*	*F*1	*Acc*	*F*1	*Acc*	*F*1	*Acc*	*F*1
*Len* < 100	0.7313	0.7305	0.7335	0.7315	0.7382	0.7372	0.7402	0.7390	0.7431	0.7427
100 ≤ *Len* < 200	0.7462	0.7454	0.7521	0.7516	0.7595	0.7572	0.7481	0.7473	0.7552	0.7542
200 ≤ *Len* < 300	0.7775	0.7763	0.7736	0.7711	0.7689	0.7664	0.7634	0.7625	0.7693	0.7675
300 ≤ *Len* < 400	0.7654	0.7642	0.7714	0.7695	0.7674	0.7669	0.7670	0.7654	0.7635	0.9618
*Len* ≥ 400	0.7532	0.7519	0.7598	0.7584	0.7560	0.7551	0.7515	0.7493	0.7543	0.7534
All	0.7571	0.7564	0.7606	0.7593	0.7585	0.7576	0.7552	0.7541	0.7587	0.7569

**Table 9:** Comparison of experimental results between different methods (IMDB dataset).

Classifier	Metrics	LDA	W2V	GLV	FPW	ETI	WWE	SFM (λ=0.5)	DFM (δ=700)
KNN	*Acc*	0.8625	0.9471	0.9368	0.9583	0.8863	0.9568	0.9685	0.9713
	*F*1	0.8619	0.9465	0.9354	0.9526	0.8847	0.9551	0.9673	0.9708
SVM	*Acc*	0.8674	0.9580	0.9476	0.9632	0.8796	0.9587	0.9661	0.9776
	*F*1	0.8623	0.9563	0.9461	0.9618	0.8772	0.9575	0.9641	0.9765

**Table 10:** Comparison of experimental results between different methods (20 Newsgroups dataset).

Classifier	Metrics	LDA	W2V	GLV	FPW	ETI	WWE	SFM (λ=0.7)	DFM (δ=800)
KNN	*Acc*	0.6635	0.7384	0.7236	0.7523	0.6812	0.7316	0.7529	0.7517
	*F*1	0.6618	0.7371	0.7219	0.7508	0.6708	0.7209	0.7516	0.7506
SVM	*Acc*	0.6724	0.7129	0.7196	0.7462	0.6951	0.7311	0.7553	0.7606
	*F*1	0.6708	0.7113	0.7183	0.7450	0.6938	0.7294	0.7532	0.7593

## Data Availability

Publicly available datasets were analyzed in this study. This data can be found here: [https://datasets.imdbws.com/, https://www.kaggle.com/crawford/20-newsgroups] accessed on 26 Janaury 2022.
